# Pulsed DPOAEs in serial measurements: Combined analysis paradigm of simultaneously occurring changes in hearing thresholds and DPOAEs

**DOI:** 10.1007/s00106-024-01478-z

**Published:** 2024-07-03

**Authors:** Katharina Bader, Dennis Zelle, Anthony W. Gummer, Ernst Dalhoff

**Affiliations:** 1 Klinik für Hals-Nasen-Ohren-Heilkunde, Universitätsklinikum Tübingen, Elfriede-Aulhorn-Straße 5, 72076 Tübingen, Germany; 2 Section of Physiological Acoustics and Communication, Universitäts-HNO-Klinik Tübingen, Tübingen, Germany; 3 Earlab GmbH, Tübingen, Germany

**Keywords:** Time domain analysis, Cochlea amplifier, Ototoxicity, Test-retest reliability, Analyse im Zeitbereich, Cochleärer Verstärker, Ototoxizität, Test-Retest-Zuverlässigkeit

## Abstract

**Background:**

To date, there is no consensus on how to standardize the assessment of ototoxicity in serial measurements. For the diagnosis of damage to the cochlear amplifier, measurement methods are required that have the highest possible test-retest reliability and validity for detecting persistent damage. Estimated distortion-product thresholds (*L*_EDPT_) based on short-pulse distortion-product otoacoustic emission (DPOAE) level maps use individually optimal DPOAE stimulus levels and allow reliable quantitative estimation of cochlea-related hearing loss.

**Materials and methods:**

Hearing thresholds were estimated objectively using *L*_EDPT_ and subjectively using modified Békésy tracking audiometry (*L*_TA_). Recordings were performed seven times within three months at 14 frequencies (*f*_2_ = 1–14 kHz) in 20 ears (PTA_4__(0.5–4_ _kHz)_ < 20 dB HL). Reconstruction of the DPOAE growth behavior as a function of the stimulus levels *L*_1_, *L*_2_ was performed on the basis of 21 DPOAE amplitudes. A numerical fit of a nonlinear mathematical function to the three-dimensional DPOAE growth function yielded *L*_EDPT_ for each stimulus frequency. For the combined analysis, probability distributions of hearing thresholds (*L*_TA_, *L*_EDPT_), DPOAE levels (*L*_DP_), and combinations thereof were determined.

**Results:**

*L*_TA_ and *L*_EDPT_ each exhibited a test-retest reliability with a median of absolute differences (AD) of 3.2 dB and 3.3 dB, respectively. Combining *L*_EDPT_, *L*_DP_, and *L*_TA_ into a single parameter yielded a significantly smaller median AD of 2.0 dB.

**Conclusion:**

It is expected that an analysis paradigm based on a combination of *L*_EDPT_, suprathreshold *L*_DP_, and fine-structure-reduced *L*_TA_ would achieve higher test performance (sensitivity and specificity), allowing reliable detection of pathological or regenerative changes in the outer hair cells.

## Abstract (de)

**Hintergrund:**

Bisher gibt es keinen Konsens darüber, wie Ototoxizität in Verlaufsmessungen standardisiert zu erfassen ist. Für die Diagnostik von Schädigungen des cochleären Verstärkers sind Messverfahren notwendig, die eine möglichst hohe Test-Retest-Zuverlässigkeit und eine hohe Aussagekraft hinsichtlich persistierender Schädigungen aufweisen. Hörschwellenschätzungen auf der Grundlage von Kurzpuls-DPOAE-Pegelkarten („estimated distortion-product thresholds“, *L*_EDPT_) berücksichtigen individuell optimale DPOAE-Anregungspegel und erlauben eine zuverlässige quantitative Schätzung des cochleär bedingten Hörverlusts.

**Methodik:**

Hörschwellen wurden mithilfe von *L*_EDPT_ objektiv geschätzt und mit einer modifizierten Békésy-Tracking-Audiometrie (*L*_TA_) subjektiv erfasst. Die Messungen wurden siebenmal innerhalb von drei Monaten bei 14 Frequenzen (*f*_2_ = 1–14 kHz) in 20 Ohren (PTA_4 (0,5–4_ _kHz)_ < 20 dB HL) durchgeführt. Die Rekonstruktion des DPOAE-Wachstumsverhaltens in Abhängigkeit von den Anregungspegeln *L*_1_,*L*_2_ erfolgte auf der Grundlage von 21 DPOAE-Amplituden und ermöglichte mithilfe einer numerischen Anpassung einer nichtlinearen mathematischen Funktion die Berechnung eines *L*_EDPT_ für jede Anregungsfrequenz. Für die gleichzeitige kombinierte Betrachtung wurden Verteilungen der Hörschwellen (*L*_TA_, *L*_EDPT_), der DPOAE-Pegel (*L*_DP_) und Kombinationen davon ermittelt.

**Ergebnisse:**

Einzeln betrachtet wiesen *L*_TA_ und *L*_EDPT_ jeweils eine Test-Retest-Zuverlässigkeit mit einem Median der absoluten Differenzen (AD) von 3,2 dB bzw. 3,3 dB auf, der sich durch Anwendung eines kombinierten Analyseparadigmas aus *L*_EDPT_, *L*_DP_ und *L*_TA_ auf 2,0 dB signifikant reduzieren ließ.

**Schlussfolgerung:**

Es ist zu erwarten, dass ein auf einer Kombination von *L*_EDPT_, überschwelligen *L*_DP_, und feinstrukturreduzierter *L*_TA_ basierendes Analyseparadigma eine höhere Güte (Sensitivität und Spezifität) des Tests erzielt, um pathologische oder auch regenerative Veränderungen der äußeren Haarsinneszellen zuverlässig zu detektieren.

To date, there is no consensus on how to standardize the assessment of ototoxicity in serial measurements. DPOAE paradigms currently used in the clinic cannot differentiate between the two DPOAE components arising from continuous sound stimulation. Nor do the paradigms take into account the individual middle-ear transfer function. Pulsed DPOAEs with individually optimal stimulus levels lead to improved validity and lower variability of test results in subjects with normal hearing. A combined analysis of pulsed DPOAEs and hearing thresholds best resolves changes in hearing status.

## Background

The aim of follow-up assessments of the functional state of the cochlear amplifier is to track changes with high sensitivity and specificity. In everyday clinical practice, serial measurements are used, for example, for the timely recognition of the influence of ototoxic substances on hearing function or to establish the efficacy of regenerative therapy. To date, however, there is no international consensus on how to assess ototoxicity or regeneration in a standardized way. The American Academy of Audiology considers the determination of the pure-tone hearing threshold, especially in the high-frequency range, and the measurement of distortion-product otoacoustic emissions (DPOAEs) to be the most reliable, clinically applicable methods [10].

DPOAEs represent intermodulation products resulting from the simultaneous stimulation of the cochlea with two stimulus tones of frequencies *f*_1_ and *f*_2_ (typically, *f*_2_*/f*_1_ *≈* 1.2) with stimulus levels *L*_1_ and *L*_2_. DPOAEs are directly based on the nonlinearity of the mechanoelectrical transduction of the outer hair cells located near the characteristic place of the stimulus frequency *f*_2_. Therefore, DPOAEs provide frequency-specific information about the functional state of the cochlear amplifier [2].

A previous recommendation for monitoring and evaluating ototoxicity in children and adolescents includes the medical history, pure-tone audiometry for the frequencies 1–8 kHz, DPOAE, and tympanometry [6]. Accordingly, a test battery of different methods should be performed, as individual methods are not sufficiently informative. Preliminary studies suggest that DPOAEs detect changes in hearing earlier than pure-tone audiometry and have a higher sensitivity to subtle or subclinical changes [7]. DPOAE thresholds showed higher sensitivity than single DPOAE levels in two studies [13, 22]. High-frequency audiometry (HFA) at 9–16 kHz can detect hearing changes more often than pure-tone audiometry [1]. In children, DPOAEs are used to detect early ototoxic, cisplatin-induced decreases in amplitude or signal-to-noise ratio (SNR) of DPOAEs [14].

In audiological follow-up examinations, a high test–retest reliability of the measurement method is essential in order to distinguish systematic pathological or regenerative changes from random measurement inaccuracies; the validity of the measurement method is equally important. For example, DPOAE level changes (< 6 dB) observed with clinically used DPOAE protocols alone cannot predict, with sufficient sensitivity and specificity, an ototoxic hearing-threshold increase verified by pure-tone audiometry [16]. Multivariate analyses that take into account DPOAE levels at neighboring frequencies, SNR, and the dose–response relationship increase the predictive power for detecting ototoxic hearing damage, but have not yet become established in clinical practice [16]. Consequently, there is currently no clinically validated, significant DPOAE change that predicts potential cochlear damage [15, 22].

DPOAEs are currently regarded in the clinic as a useful, supplementary method for the diagnosis of cochlear function, but they have limitations in their diagnostic value [11]. There are three main limiting factors: (1) DPOAEs essentially consist of two components, the nonlinear distortion component and the coherent reflection component, which are generated at different locations along the organ of Corti by different mechanisms [25]. Depending on both the level and the phase differences between the components, the waves can variously interfere and thus lead to artifact-prone measurement results [29]. (2) DPOAE signals are influenced by individual middle-ear characteristics, particularly of retrograde transmission [17]. (3) DPOAE levels show a relatively limited correlation with cochlear hearing loss, with the relationship being nonlinearly dependent on both level and frequency [4, 12].

An extended DPOAE diagnostic approach is provided by DPOAE growth functions, which semi-logarithmically map the sound pressure of the DPOAE amplitude as a function of the stimulus level *L*_2_ for each frequency. Extrapolation of a regression line to the *L*_2_ axis yields the so-called estimated distortion-product threshold (EDPT), whereby its level is denoted by *L*_EDPT_. The EDPT correlates approximately 1:1 with the hearing threshold [5]. Diagnostic precision is significantly improved by the artifact-free acquisition and analysis of DPOAEs in the time domain using pulsed stimuli [8, 27, 29, 30], together with the application of individually optimal, frequency-specific stimulus levels, which are acquired using DPOAE level maps [28]. DPOAE level maps depict the growth behavior of DPOAE amplitude as a function of stimulus-level combinations that sample an extended area in *L*_1_,*L*_2_ space and allow for the derivation of *L*_EDPT_ by numerically fitting a nonlinear mathematical function to the DPOAE amplitude samples. Importantly, this procedure does not require a priori choice of a stimulus path to yield the maximum DPOAE amplitudes for a given subject [28].

The *L*_EDPT_ provides a promising method for ascertaining true changes in the functional state of the cochlear amplifier. The *L*_EDPT_ can quantify hearing loss with high accuracy [28] and has a high test–retest reliability [3], yielding high sensitivity and specificity in serial monitoring. DPOAE levels, which predominate in studies of serial monitoring compared with DPOAE thresholds, also exhibit particularly high test–retest reliability [9, 20, 23]. However, given that the hearing threshold appears to be approximately proportional to the DPOAE level with a slope of 2 [18], then significant differences in the test–retest reliabilities of DPOAE levels and thresholds—either DPOAE thresholds or hearing thresholds—can only be estimated after first multiplying the changes of DPOAE level by 2 [3]. When comparing test–retest reliabilities, not only must the measurement times be kept in mind, but also that DPOAE levels primarily contain information about the suprathreshold behavior of the cochlear amplifier, whereas DPOAE thresholds characterize behavior near neural threshold and thus assess the maximum amount of cochlear amplification.

The aim of the present study was to reduce the influence of measurement inaccuracies in the respective methods and to increase the test–retest reliability by using a combined analysis paradigm of pure-tone threshold (*L*_TA_), estimated distortion-product threshold (*L*_EDPT_), and DPOAE level (*L*_DP_).

## Material and methods

### Study design and measurement system

For the combined analysis paradigm presented here, DPOAE level maps and hearing thresholds from a study conducted by the authors [3] were used, in which the test–retest reliability of the level map-based *L*_EDPT_ was compared with that of pure-tone thresholds. Measurements were recorded seven times over three months at 14 frequencies between 1 and 14 kHz in 20 ears of ten normal-hearing subjects (PTA_4 (0.5–4_ _kHz)_ < 20 dB HL; age 32.1 ± 9.7 years). Subjective hearing thresholds, *L*_TA_, were recorded three times at each frequency and at two adjacent frequencies using modified Békésy tracking audiometry, creating a frequency group to smooth out fine-structure effects in the behavioral audiogram. The study was approved by the Ethics Committee of the University of Tübingen (265/2018B01) and conducted in accordance with the Declaration of Helsinki for experiments with humans.

All measurements were conducted with two ER-10C probes (Etymotic Research, Elk Grove Village, IL, USA) using a standard PC with NI measurement cards (National Instruments, Austin, TX, USA). Stimulation and data acquisition were carried out using measurement software implemented in LabVIEW (National Instruments). Specially developed software in MATLAB (The MathWorks, Natwick, MA, USA) enabled automated analysis of the DPOAEs and hearing thresholds. In order to achieve consistent placement of the sound probes across all sessions, the frequency response of the ear-canal sound pressure from 0.3 to 20 kHz was determined for each ear and visually compared with that from previous sessions. The stimulus sound pressure was calibrated before each session by an in-ear measurement and the transmission to the eardrum was corrected using an artificial ear (B&K type 4157, Brüel & Kjær, Nærum, Denmark).

### DPOAE level maps

**Fig. 1:**
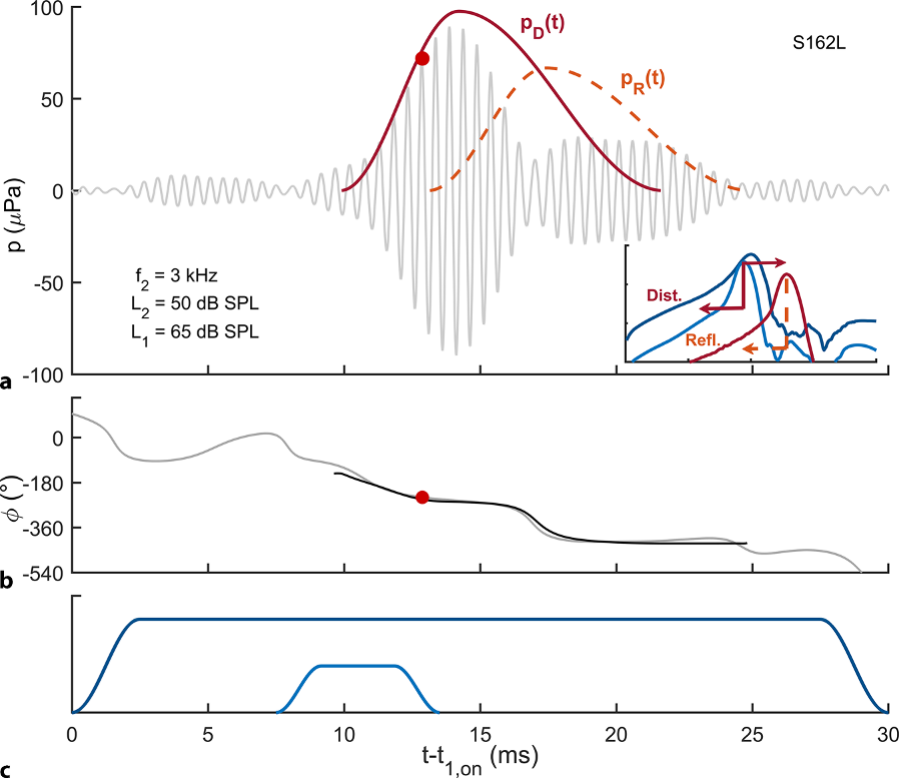
Separation of the main DPOAE components in the time domain using short-pulse stimulation. DPOAEs are generated with two pulsed stimulus tones of different lengths of frequency *f*_2_ (*light blue*) and *f*_1_ (*dark blue*); the stimulus levels are *L*_2_ and *L*_1_ (**c**, schematic diagram). The envelopes of travelling waves are sketched along the unrolled basilar membrane (inset in **a** with ordinate logarithmic over three decades). In the vicinity of the characteristic place of the *f*_2_ tone, the interaction of the *f*_2_ and *f*_1_ travelling waves produces the so-called nonlinear distortion component (*Dist.*) of frequency *f*_DP_ = 2*f*_1_ − *f*_2_, which propagates retrograde as a travelling wave in the direction of the oval window. This distortion component also propagates anterograde (*dark red*) from the *f*_2_ characteristic place to the *f*_DP_ characteristic place, where it is partially reflected from local scattering centers and then propagates retrograde in the direction of the oval window, forming the coherent reflection component (*Refl.)* of the DPOAE. The DPOAE signal is the sum of the nonlinear distortion and coherent reflection components. Time course of the DPOAE amplitude (**a**) and phase (**b**) relative to the switch-on time *t*_1,on_ of the *f*_1_ tone, recorded in ear S162L at *f*_2_ = 3 kHz, *L*_2_ = 50 dB sound pressure level (SPL), *L*_1_ = 65 dB SPL. **a** Measured DPOAE signal (*light grey line*). *Dark red dot*: Amplitude of the nonlinear distortion component, *P*_DP_. Envelope of the distortion component, *p*_D_(*t*) (*dark red line*), and reflection component, *p*_R_(*t*) (*dashed light red lin*e). **b** Instantaneous phase of the measured DPOAE signal (light gray) and instantaneous phase of the calculated DPOAE signal, *p*_D_(*t*) + *p*_R_(*t*) (*black*). Thus, the DPOAE essentially consists of two components that are generated at two different locations in the cochlea by two different mechanisms. Depending on the relative amplitudes and phases between the components, the waves interfere; for example, as shown here in the time signal (**a**), a phase difference near 180° (**b**) causes almost mutual cancellation of the two components. Cancellation would lead to the incorrect conclusion that the cochlear amplifier has been damaged. Since the DPOAE components have different latencies, the two DPOAE components can be separated by stimulating with short-pulsed tones and extracting the nonlinear distortion component in the time domain, thus avoiding an erroneous conclusion about the functional state of the cochlear amplifier. For a detailed illustration of short-pulse stimulation, we refer the interested reader to Zelle et al. (2016) [29]

For the bilateral acquisition of DPOAE level maps, measured simultaneously from the two ears, 21 short-pulse DPOAEs of different stimulus levels (*L*_1,_*L*_2_ pairs) were acquired at each frequency pair (*f*_2_ = 1–14 kHz, *f*_2_/*f*_1_ = 1.2). The short-pulse stimulation enables the separation of the nonlinear distortion and coherent reflection components in the time domain by utilizing their different latencies (Fig. 1).

The artifact-free DPOAE amplitude, *P*_DP_ (red dot in Fig. 1a), extracted from the time signal, estimates the amplitude of the nonlinear distortion component; it was accepted for statistical analysis for SNR ≥ 10 dB and converted to the DPOAE level, *L*_DP_. The total measurement time for all DPOAE level maps with 21 levels at 14 frequencies in both ears was 12.6 min.

**Fig. 2:**
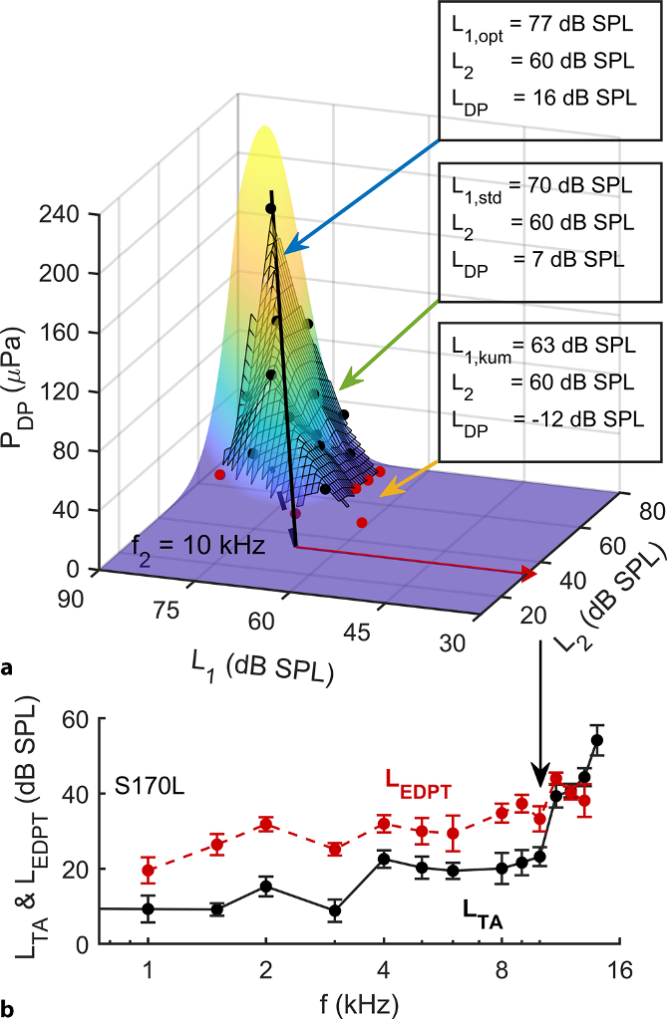
**a** Individual model level map reconstructed from accepted DPOAE amplitudes *P*_DP_ (*black dots*) recorded for ear S170L at *f*_2_ = 10 kHz. *Red dots*: *P*_DP_ with SNR < 10 dB. *Solid black line*: Ridge representing the optimal semi-logarithmic growth function. *Red arrow*: Estimated distortion-product threshold using the DPOAE level map; *L*_EPDT_ = 33.25 ± 3.34 dB SPL. In this example, at *L*_2_ = 60 dB SPL, the stimulus levels *L*_1,std_ = 70 dB SPL (*green arrow*) and *L*_1,kum_ = 63 dB SPL (*yellow arrow*) would be suboptimal and would generate significantly reduced *P*_DP_ or DPOAE levels (*L*_DP_) compared with the individually optimal stimulus level *L*_1,opt_ = 77 dB SPL (*blue arrow*). **b** EDPT-gram *L*_EDPT_ (red) and the audiogram *L*_TA_ (black) for *f* = 1–14 kHz. The course of the EDPT-gram shows a high correlation with the subjective tone-threshold audiogram

Plotting of the measured DPOAE amplitudes as a function of the stimulus levels *L*_1_ and *L*_2_ enables construction of a DPOAE level map (Fig. 2, three-dimensional surface). The map allows an individually optimal growth function to be determined (Fig. 2, black line); that is, a set of DPOAE amplitudes, specific for a given frequency pair and subject, which are maximal for a given *L*_2_. This growth function was obtained by numerically fitting a mathematical function to the measured DPOAE amplitudes. An individual, frequency-specific stimulus level *L*_1,opt_ for a given *L*_2_ is determined from projection of the optimal growth function in the *L*_1_,*L*_2_ plane. *L*_EDPT_ is given as the value of *L*_2_ at which the optimal growth function intersects the *L*_1_,*L*_2_ plane (Fig. 2, red arrow). With this method, *L*_EDPT_ can be determined without having to define optimal stimulus levels in advance of the experiment [28].

In addition, the model level maps can be used to reconstruct those *L*_DP_ that would have been generated with the frequency-independent stimulus paradigm proposed by Kummer et al. [18]; namely, *L*_1,kum_ = 0.4*L*_2_ + 39 dB. The maps can also be employed to reconstruct those *L*_DP_ that would have been generated with the constant level-spacing paradigm conventionally used as a clinical standard; namely, *L*_1,std_ = *L*_2_ + 10 dB.

In this work, reconstructed *L*_DP_ are presented exemplarily for *L*_2_ = 45 and 65 dB sound pressure level (SPL). It is important to realize that the reconstructed *L*_DP_ only provide meaningful estimates of actual measured values to the extent that the model function faithfully reproduces the actual dependence on the stimulus-level combinations. For this reason, as well as due to the usual limiting conditions of practical measurability (usually residual noise level < −20 dB SPL [4, 12]), reconstructed *L*_DP_ < −15 dB SPL were not evaluated and therefore excluded from further analysis.

### Combined analysis

For the purpose of developing an analysis paradigm that combines simultaneously occurring changes in *L*_DP_, *L*_EDPT_, and *L*_TA_ from examination to examination, dependencies between these parameters must be assigned. It is known that *L*_EDPT_ and *L*_TA_ are correlated approximately with a ratio of 1:1 [28, 30]; i.e., an increase in hearing threshold by 10 dB is accompanied by an increase in *L*_EDPT_ of also about 10 dB. For the dependency of *L*_DP_ and *L*_TA_, we use the observation made by Kummer et al. (see their Fig. 7b and their Tables II, III; [18]) that an increase in hearing threshold by 10 dB was associated with a decrease in *L*_DP_ of only about 5 dB; i.e., correlated with a ratio of approximately 2:1. Overall, this observation means that in order to estimate an increase of a cochlear-induced hearing threshold from a decrease of *L*_DP_, the *L*_DP_ decrease would have to be doubled. Therefore, in a combined analysis, a change in *L*_DP_ was weighted by a factor of (negative) 2. The analysis investigated the following four combinations of the changes ∆*L*_TA_, ∆*L*_DP,_ and ∆*L*_EDPT_: namely, (∆*L*_TA_ − 2∆*L*_DP_)/2, (∆*L*_TA_ + ∆*L*_EDPT_)/2, (∆*L*_EDPT_ − 2∆*L*_DP_)/2 and (∆*L*_TA_ + ∆*L*_EDPT_ − 2∆*L*_DP_)/3.

### Statistical analysis

The software SPSS Statistics (version 26, IBM Corp., Armonk, NY, USA) was used for the statistical tests. To quantify the test–retest reliability of the *L*_EDPT_, *L*_TA_, and *L*_DP_, the absolute differences (AD) between two visits (1 vs. 2, 1 vs. 3, 1 vs. 4, …, 2 vs. 3, 2 vs. 4, …; *N* = 21) were used as a metric [20, 21]. Test–retest reliability determines the ability of a method to produce similar results when repeated for the same individual under the same experimental conditions. The statistical significance of the absolute differences between the samples was tested using the Friedman test.

## Results

### DPOAE level

**Fig. 3:**
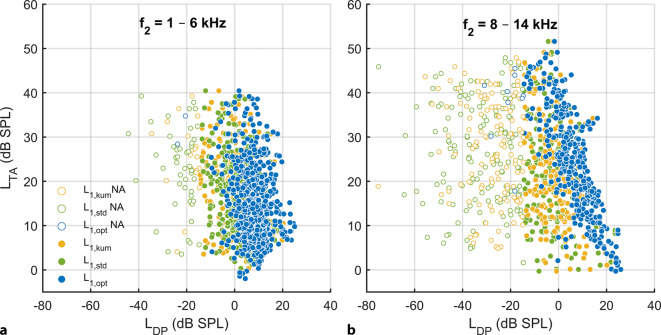
Subjective hearing threshold *L*_TA_, recorded using modified Békésy tracking audiometry, as a function of DPOAE level *L*_DP_, reconstructed using model DPOAE level maps. The stimulus levels were chosen as follows: *L*_2_ = 45 dB SPL and *L*_1_ chosen with three strategies: *L*_1,opt_: individually optimized, frequency-specific stimulus level (*blue*), *L*_1,std_ = *L*_2_ + 10 dB (*green*) and *L*_1,kum_ = 0.4*L*_2_ + 39 dB (*yellow*). *L*_DP_ ≤ −15 dB SPL are considered statistically unacceptable and are represented by *open circles*. **a** Frequency range *f*_2_ = 1–6 kHz, *N* = 649/980. **b** Frequency range *f*_2_ = 8–14 kHz, *N* = 353/980

The correlation between *L*_DP_ and *L*_TA_ depended significantly on the stimulus levels (Fig. 3). For *f*_2_ = 8–14 kHz, in which there was already a high-frequency hearing loss in some subjects and thus a larger *L*_TA_ range, there was a strong correlation between *L*_DP_ and *L*_TA_ (Spearman’s *ρ* = −0.737; *p* < 0.001) when *L*_DP_ was reconstructed using individually optimal, frequency-specific stimulus levels *L*_1,opt_ (blue) at *L*_2_ = 45 dB SPL (Table 1). By contrast, when *L*_DP_ was reconstructed with frequency-independent, standard clinically used stimulus levels *L*_1,std_ (green, *L*_1_ = *L*_2_ + 10 dB) or *L*_1,kum_ (yellow, *L*_1_ = 0.4*L*_2_ + 39 dB), there was only a small correlation between *L*_DP_ and *L*_TA_ (*L*_1,std_ Spearman’s *ρ* = −0.202, *p* = 0.003; *L*_1,kum_ Spearman’s *ρ* = −0.282, *p* < 0.001). For *f*_2_ = 1–6 kHz, there was slight hearing loss and therefore insufficient *L*_TA_ range to establish a potential correlation between *L*_DP_ and *L*_TA_. The stimulus level *L*_2_ = 45 dB SPL is shown as an example in Fig. 3 and Table 1 because the cochlear amplifier is not yet in compression at this level and a sufficient number of detectable DPOAE signals were generated.

**Table 1:** Linear regression analysis of the dependent variable hearing threshold *L*_TA_ (dB SPL) and the independent variable DPOAE level *L*_DP_ (dB SPL) for the different excitation paradigms and frequency ranges (*L*_2_ = 45 dB SPL); data are shown in Fig. 3. For *f*_2_ = 8–14 kHz, the quality of the estimation, quantified by the standard deviation *σ* (dB) of the estimate, is significantly higher for *L*_1_,_opt_ compared with *L*_1_,_std_ and *L*_1_,_kum_. *r*^*2*^: correlation coefficient squared

*f*_2_		y =	*r*^2^	σ	*p*	*N*
1–6 kHz	*L*_1,opt_	16.57–0.268*x*	0.030	8.05	< 0.001	649
	*L*_1,kum_	16.57–0.479*x*	0.136	7.43	< 0.001	631
	*L*_1,std_	15.81–0.484*x*	0.138	7.24	< 0.001	605
8–14 kHz	*L*_1,opt_	30.62–1.079*x*	0.601	7.13	< 0.001	353
	*L*_1,kum_	22.41–0.479*x*	0.125	10.38	< 0.001	240
	*L*_1,std_	21.77–0.358*x*	0.071	10.69	< 0.001	215

**Fig. 4:**
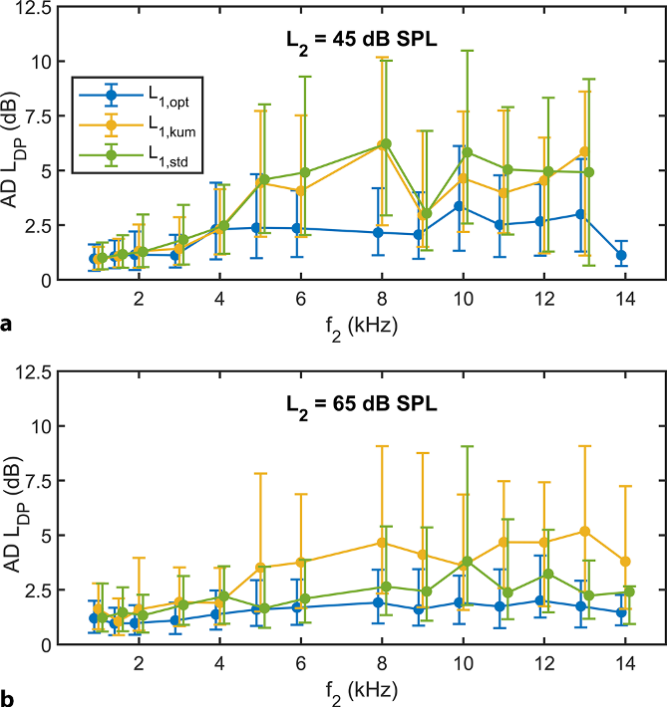
Test–retest reliability of the DPOAE level *L*_DP_ demonstrated using the median of the absolute differences (AD) for the individual frequencies as a function of the stimulus levels *L*_1,opt_ (*blue*), *L*_1,kum_ = 0.4*L*_2_ + 39 dB (*yellow*) and *L*_1,std_ = *L*_2_ + 10 dB (*green*). The *lower error bar* corresponds to the 25th percentile, the *upper error bar* to the 75th percentile. The graphs have been slightly offset along the abscissa for better readability. **a**: *L*_2_ = 45 dB SPL. **b**: *L*_2_ = 65 dB SPL

The test–retest reliability of *L*_DP_ increased significantly with individually optimized stimulus levels (*L*_1,opt_; Fig. 4). The median AD of *L*_DP_ decreased significantly from 2.3 dB (*L*_1,std_) or 2.2 dB (*L*_1,kum_) to 1.8 dB using *L*_1,opt_ at *L*_2_ = 45 dB SPL (Table 2, Friedman test, *F* = 734.65; *p* < 0.0001). Consequently, the derived reference interval decreased, respectively, from 10 or 9 dB to 6 dB, defined here as the 90th percentile of AD. At *L*_2_ = 65 dB SPL, the median AD using *L*_1,opt_ was reduced from 2.4 dB (*L*_1,kum_) or 1.9 dB (*L*_1,std_) to 1.4 dB and the reference interval decreased, respectively, from 9 or 7 dB to 4 dB (Table 2).

**Table 2:** Test–retest reliability of the DPOAE level *L*_DP_ as a function of stimulus level for *f*_2_ = 1–14 kHz, shown using the median, the interquartile range (IQR), and the 90th percentile of the absolute differences (dB) for *L*_2_ = 45 and 65 dB SPL and in each case for *L*_1_,_opt_, *L*_1,kum_ = 0.4*L*_2_ + 39 dB, and *L*_1_,_std_ = *L*_2_ + 10 dB. The test–retest reliability of *L*_DP_ was significantly increased by selecting individually optimized, frequency-specific excitation levels *L*_1_,_opt_ at *L*_2_ = 45 and 65 dB SPL (Friedman test, *F* = 383.90/482.37, *p* < 0.0001)

		Absolute difference (AD) of DPOAE level (*L*_DP_)						
	*L*_2_ = 45 dB SPL				*L*_2_ = 65 dB SPL			
	Median	IQR	90%	*N*	Median	IQR	90%	*N*
*L*_1,opt_	1.8	2.8	6.2	2469	1.4	1.9	4.1	2508
*L*_1,kum_	2.2	4.0	8.9	2123	2.4	4.2	9.4	2345
*L*_1,std_	2.3	4.3	9.5	1991	1.9	2.9	7.0	2499

### Estimated hearing thresholds based on DPOAE level maps

**Fig. 5:**
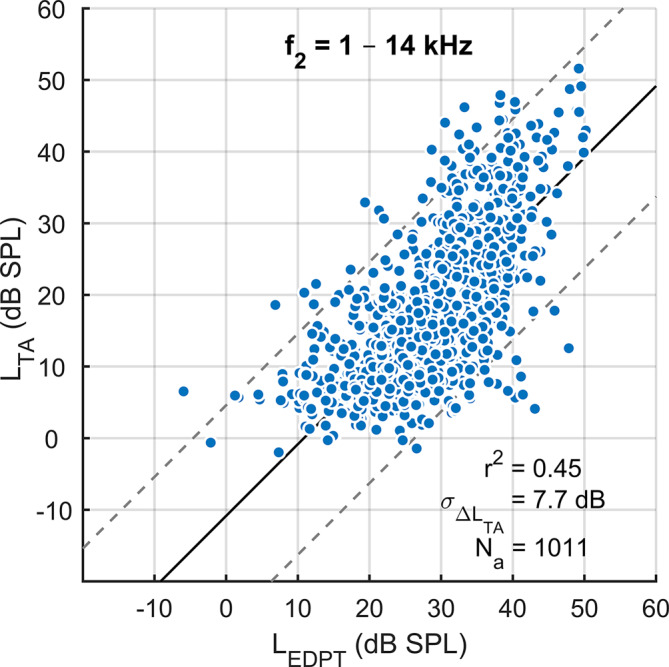
Correlation of the subjective hearing thresholds *L*_TA_ recorded using modified Békésy tracking audiometry, with DPOAE-based hearing threshold estimates *L*_EDPT_, derived from DPOAE level maps. Data from all frequencies, subjects, and sessions have been pooled. *Solid black line *and *dashed gray lines* represent, respectively, the regression line and the 95% confidence intervals. $\sigma _{{\Updelta L_{\mathrm{TA}}}}$ = 7.7 dB is the standard deviation (SD) of *L*_TA_ from the regression line. *N*_a_ = 1011/1960 is the number of accepted *L*_EDPT_

**Fig. 6:**
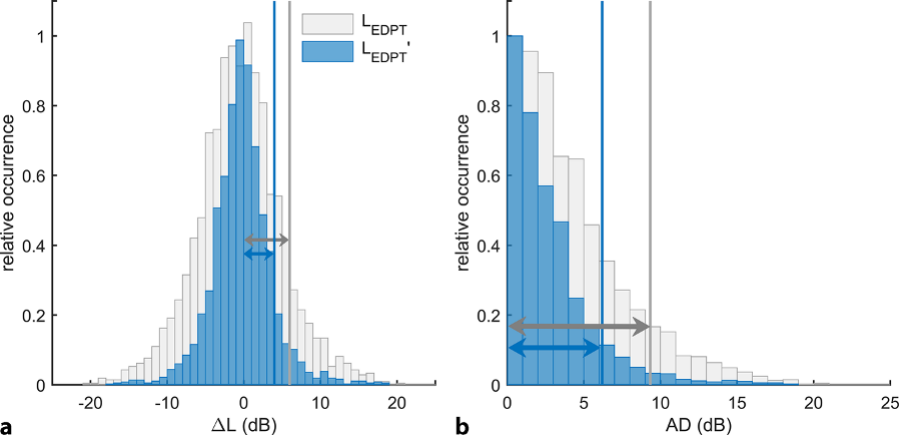
**a** Test–retest reliability of an *L*_EDPT_ based solely on DPOAE level maps compared with the combined measure *L*_EDPT_′ = (∆*L*_EDPT_ + ∆*L*_TA_ − 2∆*L*_DP,opt,65_)/3, shown using the normalized histograms of the differences between two sessions pooled across all subjects and frequencies *f*_2_ = 1–14 kHz. *N* = 2345/5880. *L*_EDPT_′ shows a clear reduction in outliers compared with *L*_EDPT_ and a reduced standard deviation from 5.6 dB (*gray arrow*) to 3.9 dB (*blue arrow*). **b** Histograms of the absolute differences (AD) of *L*_EDPT_ (grey) and *L*_EDPT_′ (*blue*). *The vertical lines* mark the reference range, defined by the 90th percentile. Using the combined measure, the reference range is reduced from 9.3 to 6.2 dB, which means that actual changes in the hearing threshold can be detected much earlier

*L*_EDPT_ and *L*_TA_ are correlated linearly (*L*_TA_ = 0.86*L*_EDPT_ − 6.7 dB, *r*^2^ = 0.45, SD = 7.7 dB, *p* < 0.001, Fig. 5). In addition to the high correlation with *L*_TA_, *L*_EDPT_ also showed a high test–retest reliability, with a median AD of 3.3 dB for *f*_2_ = 1–14 kHz (Table 3). Table 3 quantifies the test–retest reliability using the median, the interquartile range (IQR), and the 90th percentile of AD for *L*_TA_ and *L*_EDPT_ as well as for different combined analysis paradigms. The combination of *L*_EDPT_, *L*_TA_, and *L*_DP,opt,65_ (*L*_1,opt_ at *L*_2_ = 65 dB SPL), denoted by *L*_EDPT_′, presented the smallest median AD (2.0 dB). An almost equally low median AD (2.1 dB) was observed when both DPOAE measures (*L*_DP_ and *L*_EDPT_) were combined. Figure 6a shows the distributions of differences between two sessions for each of *L*_EDPT_ and *L*_EDPT_′. The combined measure, *L*_EDPT_′, presents a significant reduction in the number of outliers compared with *L*_EDPT_ and a reduction in the standard deviation from 5.6 to 3.9 dB (vertical lines). Figure 6b shows the distributions of absolute differences (AD) for *L*_EDPT_ and *L*_EDPT_′. The vertical lines mark the reference range, defined by the 90th percentile. The reference range of *L*_EDPT_′ (6.2 dB) is significantly smaller than that of *L*_EDPT_ (9.3 dB). Taken together, these observations imply that the combined measure *L*_EDPT_′ presents significantly higher test–retest reliability than *L*_EDPT_.

**Table 3:** Test–retest reliability of hearing thresholds (*L*_TA_) using modified Békésy tracking audiometry, DPOAE-based estimated hearing thresholds (*L*_EDPT_), and the combined measures for the frequency range *f*_2_ = 1–14 kHz, presented using the median, interquartile range (IQR), and the 90th percentile of absolute differences (AD). The test–retest reliability can be significantly reduced by the combined measures (Friedman test, *F* = 788.52, *p* < 0.0001)

Metric	AD (dB)			
	Median	IQR	90%	*N*
∆*L*_TA_	3.2	4.7	10.2	5607
∆*L*_EDPT_	3.3	4.4	9.3	2508
(∆*L*_EDPT_ + ∆*L*_TA_) / 2	2.5	3.5	7.6	2503
(∆*L*_TA_ − 2∆*L*_DP,opt,65_) / 2	2.3	3.1	7.1	2503
(∆*L*_EDPT_ − 2∆*L*_DP,opt,65_) / 2	2.1	3.1	6.3	2508
(∆*L*_EDPT_ + ∆*L*_TA_ − 2∆*L*_DP,opt,65_)/3	2.0	2.9	6.2	2503

## Discussion

### DPOAE level: significance and test–retest reliability

In general, DPOAE levels show a relatively limited correlation with cochlear-induced hearing loss, and the complex relationship between DPOAE level and the associated hearing loss is nonlinearly dependent on stimulus level and frequency [4, 12]. Interestingly, after using the individually optimal stimulus level *L*_1,opt_ at a moderate stimulus level *L*_2_ = 45 dB SPL, a significantly higher correlation of *L*_DP_ and *L*_TA_ and a lower scatter was found, especially in the high-frequency range *f*_2_ = 8–14 kHz (Fig. 3). Since the inclusion criterion for the study was defined as PTA_4 (0.5–4_ _kHz)_ < 20 dB HL, there was hardly any hearing loss for some frequencies in the range 1–6 kHz and, therefore, the *L*_TA_ range was insufficient to detect potential correlation between *L*_DP_ and *L*_TA_ and consequently any associated middle-ear or noise influences.

The idea behind the use of optimal stimulus levels is to achieve an ideal overlap between the travelling-wave envelopes of the two stimulus tones *f*_1_ and *f*_2_ near the characteristic place of the *f*_2_ tone by taking into account the different compression states of the two travelling waves near the *f*_2_ characteristic place [24], so that the distortion produced by nonlinear mechanoelectrical transduction is maximal near that place [2]. Although these two aims were originally introduced by Kummer et al. [18], the algorithms for attaining an optimal *L*_1_ for a given *L*_2_ differ significantly: In our case, the optimization parameters are derived individually for each session, subject, and *f*_2_ and, as such, our algorithm is a major development of the earlier optimizing algorithm (the “scissor’s” algorithm) where the parameters were independent of the subject and *f*_2_ [18]. Individually optimizing *L*_1_ led to a reduction in the inter-subject variability of *L*_DP_, especially for *f*_2_ = 8–14 kHz (Fig. 3b).

Not only the inter-subject variability of *L*_DP_, but also the intra-subject variability, the so-called test–retest reliability, improved significantly by selecting frequency-specific, individually optimal stimulus levels. Consequently, the frequently quoted reference range for an intra-subject DPOAE change from examination to examination at the stimulus level of *L*_2_ = 65 dB SPL was reduced from approximately 6–8 dB to 4–5 dB. In future clinical examinations, pulsed DPOAE signals excited using frequency-specific, individually optimal stimulus levels could therefore be a valuable method for detecting early signs of changes in the functional state of the cochlear amplifier, before they become visible with conventional DPOAE level measurements.

### Estimated DPOAE thresholds: significance and test–retest reliability

DPOAE level maps capture—with high precision—the intensity behavior of the cochlear amplifier near the *f*_2_ place for different stimulus level pairs *L*_1_,*L*_2_. *L*_EDPT_ derived from such maps incorporate information from multiple DPOAE amplitudes and thus allow for a more precise and extended diagnosis of the functional state of the cochlear amplifier, as has already been shown by us and other authors for DPOAE growth functions and their properties [26]. With numerical extrapolation of the DPOAE amplitudes, the growth behavior of the DPOAE amplitudes can also be derived at low stimulus levels. It is precisely at such levels that the test significance of the functional state of the cochlear amplifier is highest due to nonlinear amplification being highest at low stimulus levels [18].

In addition, provided that there is no damage to the inner hair cells and neural pathways, *L*_EDPT_ enables an objective quantification of the hearing threshold [8, 28, 30]. Such quantification is not possible using conventional protocols in the clinic, where suprathreshold DPOAEs are measured at one or two stimulus levels. Moreover, changes in the individual transmission characteristics of the middle ear can be captured by DPOAE level maps, whereby losses in anterograde middle-ear transmission shift and distort the DPOAE level maps and losses of retrograde middle-ear transmission reduce DPOAE amplitude [19]. The DPOAE growth function is extrapolated along the ridge of the three-dimensional surface of the model DPOAE level map (Fig. 2) and is thus based on maximum DPOAE amplitudes generated using individual, near-ideal stimulus levels. *L*_EDPT_ based on DPOAE level maps estimate hearing thresholds more accurately than conventional DPOAE growth functions that are excited with predetermined stimulus levels [28]. In this study, *L*_TA_ for *f*_2_ = 1–14 kHz correlated with the *L*_EDPT_ derived from DPOAE level maps with a standard deviation of 7.7 dB (Fig. 5). This error estimate is slightly higher than the standard deviation of 6.5 dB reported by Zelle et al. [30], and is attributable to a factor four reduction in the averaging time per DPOAE signal in the present study together with the extension of the frequency measurement range from 1–8 kHz to 1–14 kHz. In addition, for reasons of the still limited quantity of hearing-loss data, the regression analysis has not been performed at single frequencies, in which case the standard deviation of the estimate of *L*_TA_ predicted from *L*_EDPT_ for a given frequency can be significantly reduced [30]. Moreover, it is expected that the implementation of a modern calibration procedure such as IPL (integrated pressure level) or FPL (forward pressure level) would further reduce the standard deviation, particularly at high frequencies [20].

*L*_EDPT_ not only estimate individual hearing thresholds accurately, but are also stable for follow-up measurements in a given ear [3]. The test–retest reliability of *L*_EDPT_ for the entire frequency range *f*_2_ = 1–14 kHz with a median AD of 3.3 dB is comparable to that of *L*_TA_ (median AD = 3.2 dB), whereas for the high-frequency range, *f*_2_ = 11–14 kHz, *L*_EDPT_ are superior to *L*_TA_ [3]. The reference range corresponding to the 90th percentile, above which an ear must be considered in need of control in follow-up examinations, is approximately 10 dB for both *L*_EDPT_ and *L*_TA_ for *f*_2_ = 1–14 kHz. When *L*_DP_ differences are doubled to correct for *L*_TA_ being proportional to *L*_DP_ with a slope of 2, *L*_DP_ show a comparable test–retest reliability; namely, with a median AD of 2.8–3.6 dB and a 90th percentile of 8–12 dB when using *L*_1,opt_ (Table 2). Since *L*_DP_ and *L*_EDPT_ are partly subject to different confounding factors (e.g., middle-ear pathology, noise sources) and physiological mechanisms, the strategy introduced in this study was to combine the two DPOAE parameters and the auditory threshold parameter into a single parameter that is as sensitive and reliable as possible.

### Combined analysis paradigm: significance and test–retest reliability

To date, changes in the pure-tone hearing threshold and DPOAE level (typically, measured at *L*_2_ = 65 dB SPL, *L*_1,std_ = 75 dB SPL) have generally been considered separately in everyday clinical practice for the monitoring of ototoxicity. To the best of our knowledge, the test–retest reliability of concurrent changes in hearing thresholds and DPOAE levels has not been reported in the literature. Only multivariate statistical DPOAE analyses that consider DPOAE level and SNR simultaneously have been presented for predicting hearing threshold [11] and ototoxic hearing loss [16]. As predictors of hearing status, multivariate DPOAE analyses achieve better test quality compared with univariate approaches using either DPOAE level or SNR. However, even with multivariate analyses, there is still considerable overlap between the distributions for normal-hearing and hearing-impaired people, which was found to be more pronounced for the frequency range 0.75–3 kHz than for 4–8 kHz [11]. Multivariate DPOAE analyses also lead to improved test performance for predicting an increase of ototoxic-induced hearing threshold, but only when the cumulative cisplatin dose is included in the analysis [16]. Using a 6-dB change in the DPOAE level as a metric allows for little to no improvement over an analysis based on cumulative cisplatin dose and pre-exposure hearing threshold [16]. The analysis paradigm presented here, which combines changes in *L*_EDPT_, suprathreshold *L*_DP_, and fine-structure-reduced *L*_TA_, significantly improved the test–retest reliability (Fig. 6 and Table 3). It is expected that this approach will lead to higher sensitivity and specificity in future studies for detecting pathological or regenerative changes in the outer hair cells.

Since this study focused on the validation of the methodology of pulsed DPOAEs in follow-up measurements in normal-hearing subjects, there are few data for mild-to-moderate hearing loss for *f*_2_ = 1–6 kHz. Therefore, for the purpose of specifying a metric that combines the various parameters, we assumed that *L*_DP_ is correlated with *L*_TA_ with a ratio of 1:2, the assumption being mainly based on the findings of Kummer et al. [18]. Given the nonlinear dependence of *L*_DP_ on stimulus frequency and level, future applications of the combined analysis paradigm should quantify the relationship between *L*_DP_ and *L*_TA_ as a function of frequency and level using individually optimal stimulus levels *L*_1_.

Although it was shown here that the combined analysis paradigm together with the pulsed DPOAE protocol yields a higher test–retest reliability than reported to date, it still has to be established that, for example, ototoxic hearing impairment in follow-up examinations of patients receiving chemotherapy with cisplatin can be detected earlier and more sensitively by using DPOAE level maps and combined analysis paradigms compared with other audiological test procedures.

In addition, the procedure could be optimized through further technical adjustments. A modern calibration procedure for sound pressure could be implemented that avoids erroneous stimulus levels due to standing waves within the auditory canal and thus facilitates the detection of DPOAEs in still higher numbers and quality. It would also be advantageous to develop an adaptive algorithm that enables the detection of DPOAE level maps within an *L*_1_,*L*_2_ space depending on the SNR, in order to reliably construct DPOAE level maps in as many patients with residual cochlear hearing as possible in a time-efficient manner.

## Outlook

Objective hearing threshold estimates based on artifact-free short-pulse DPOAE level maps are promising for the early and sensitive detection of hearing loss, such as for ototoxicity, due to their high test–retest reliability and direct association with the functional state of the cochlear amplifier. They allow for a simple, time-efficient interpretation of the measurement results, as they represent hearing threshold estimates that are directly comparable with conventional estimates from pure-tone audiometry. The simultaneous observation of changes in DPOAE levels together with subjectively and objectively determined hearing thresholds occurring within an ear using the combined analysis paradigm presented here reduces the influence of measurement inaccuracies of the respective methods. Thus, pathological or regenerative changes in the functional state of the cochlear amplifier could potentially be detected much earlier than with conventional hearing tests. This should enable earlier interventions and potentially better treatment outcomes for patients.

## Practical conclusion

- Conventional DPOAE methods in follow-up studies do not yet allow for clinical validation of DPOAE change that can predict potential cochlear damage, such as for ototoxicity.
- In follow-up studies, the significance and test–retest reliability of the pulsed DPOAE levels depend crucially on the choice of stimulus levels and their deviation from the individually optimal stimulus.
- DPOAE level maps based on pulsed DPOAEs enable precise estimation of hearing threshold, taking into account interference effects of the DPOAE components as well as the individual middle-ear transfer function.
- Using a combined analysis paradigm, such as the one presented here, it is expected that the reliability for detecting a change in the functional state of the cochlear amplifier will be vastly improved compared with conventional DPOAE methods.

## Declarations

For this article no studies with animals were performed by any of the authors. All studies mentioned were in accordance with the ethical standards indicated in each case.

The supplement containing this article is not sponsored by industry.
